# Which patients recur as atrial tachycardia rather than atrial fibrillation after catheter ablation of atrial fibrillation?

**DOI:** 10.1371/journal.pone.0188326

**Published:** 2017-11-16

**Authors:** Pil-Sung Yang, Young-Ah Park, Tae-Hoon Kim, Jae-Sun Uhm, Boyoung Joung, Moon-Hyoung Lee, Hui-Nam Pak

**Affiliations:** 1 Yonsei University Health System, Seoul, Republic of Korea; 2 Division of Cardiology, Inje University, Busan Paik Hosipital, Busan, Republic of Korea; Indiana University, UNITED STATES

## Abstract

**Introduction:**

The ablation gaps have been known as the main mechanism of recurrence as atrial tachycardia (AT) rather than atrial fibrillation (AF) after AF catheter ablation. However, AF organization due to reduction of critical mass or focal trigger may also be the mechanism of AT recurrence. We sought to find out the main clinical factors of recurrence as AT rather than AF after AF ablation in the absence of antiarrhythmic drug effect.

**Methods:**

We analyzed 521 patients (70.8% men, 64.1% paroxysmal AF) who experienced AT or AF recurrence without antiarrhythmic drug effect during 44.7 ± 25.4 months follow-up.

**Results:**

Among 521 patients with recurrence, 42.0% (219 of 521) recurred with AT. The proportion of AT recurrence was not different between the pulmonary vein isolation only group and additional linear ablation group (45.1% vs. 38.1%, p = 0.128). The absence of hypertension (odds ratio [OR] 0.49, 95% confidence interval [CI] 0.29–0.83, p = 0.007), small left atrial (LA) volume index (OR 0.89 per 10 mL/m^2^, 95% CI 0.79–1.00, p = 0.049), and high mean LA bipolar voltage (OR 2.03 per 1 mV, 95% CI 1.30–3.16, p = 0.002) were independently associated with AT recurrence, whereas additional linear ablation was not. Among 90 patients who underwent repeat ablation procedure, rates of PV reconnection (p = 0.358) and gap in prior linear ablations (p = 0.269) were not significantly different between AT recurrence group and AF recurrence group.

**Conclusion:**

The degree of LA remodeling is significantly associated with recurrence as AT after AF ablation, irrespective of potential ablation gap in linear lesion.

## Introduction

Atrial fibrillation (AF) is one of the most commonly diagnosed cardiac arrhythmias, which is associated with diverse disease mechanisms and comorbidities, most importantly with the degenerative process associated with aging. Symptomatic patients with drug-refractory AF are proven candidates for rhythm control with radiofrequency catheter ablation (RFCA). Although RFCA is very effective in controlling AF rhythm, a significant number of patients develop recurrence,[1] and some of patients develop organized atrial tachycardia (AT) rather than AF after RFCA of AF.[2, 3] However, it is unclear whether technical failure or AF progression is the distinctive mechanism of recurrence,[4] and which clinical factors are the main determinants of recurrence patterns (AT or AF). Previous studies suggested that a small electrical gap along the ablation line results in a conduction delay in the atrium, which generates an excitable gap that might present as stable macro-reentrant AT, and that incomplete conduction block of linear ablation increases the risk of recurrent AT.[5] However, atrial tissue size and remodeling may also affect the type of atrial arrhythmias. Haissaguerre et al.[6, 7] suggested that a stepwise approach to persistent AF may increase the AF cycle length, organize the AF pattern, and finally cause the transformation of AF to AT, followed by termination of arrhythmia. Critical mass reduction by linear ablation may reduce wavebreak and inhibit AF maintenance,[8] eventually resulting in recurrence as AT instead of disorganized AF even though there was no electrical gap in linear ablation lesions. Atrial structural remodeling secondary to AF disease progression has also been suggested as a determinant of recurrence type after AF ablation.[9] Another important factor in recurrence type after AF ablation is focal trigger.[10] Lastly, antiarrhythmic drugs, especially sodium channel blockers, can influence the type of recurrence, too.[11] Therefore, the aim of this study was to investigate what clinical factors are the main determinants of recurrence patterns (AT vs. AF) after AF ablation, in the absence of antiarrhythmic drug effect.

## Methods

### Study population

The study protocol adhered to the principles of the Declaration of Helsinki and was approved by the Institutional Review Board at the Yonsei University Health System. All patients provided written informed consent for inclusion in the Yonsei AF Ablation Cohort Database (Clinicaltrials.gov; NCT02138695). Between March 2009 and December 2016, a total of 2,158 patients underwent RFCA for AF, and 1,023 consecutive patients with AF who experienced early or late recurrence after RFCA were screened from this cohort. According to published guidelines,[12] episodes of AT or AF lasting 30 seconds or more after catheter ablation of AF were considered as recurrence. Among the 1,023 patients, 521 patients (369 [70.8%] men, 334 [64.1%] paroxysmal AF) who were not receiving antiarrhythmic drugs at the time of recurrence were included in this study after excluding 502 patients with following criteria: 1) those with history of prior RFCA or cardiac surgery (n = 140), 2) those with valvular AF (moderate to severe mitral stenosis, any mechanical or bioprosthetic heart valve, or mitral valve repair; n = 43), 3) those with potential antiarrhythmic drug effect at the time of recurrence (n = 282), and 4) those who both types of recurrence (AT and AF) were documented after RFCA (n = 37).

### Electrophysiologic mapping and CT measurement of the left atrium

Details regarding electrophysiologic mapping and the RFCA technique and strategy were performed as described in previous studies.[9, 13] The left atrial (LA) voltage map was generated during de novo RFCA by recording contact bipolar electrograms from 350–500 points on the LA endocardium during right atrium pacing at a constant cycle length of 500 ms. LA voltage values were obtained by experienced operators only at secure endocardial contact points, and contact artifacts and noises were excluded in the LA voltage analysis. The mean LA voltage was calculated as described previously.[9] The three-dimensional (3D) spiral CT images of the LA were analyzed before the de novo procedure in an imaging processing workstation (Aquarius, Terarecon Inc., Foster City, CA, USA). LA images were subdivided into compartments according to the embryological origin as follows: venous LA, anterior LA, and LA appendage.

### Radiofrequency catheter ablation

We used an open irrigated-tip catheter (Celsius, Johnson & Johnson Inc., Diamond Bar, CA, USA; Coolflex, St. Jude Medical Inc., Minnetonka, MN, USA; 30–35 W; 42°C) to deliver radiofrequency (RF) energy for ablation. The ablation RF power setting was 30–35 W; however, it was 25–30 W for the posterior wall to prevent potential complications. All patients initially underwent circumferential pulmonary vein isolation (CPVI) and the cavotricuspid isthmus (CTI) ablation. The end-point of CPVI was entrance and exit block of pulmonary vein (PV) conduction. For patients with persistent AF, we added a roof line, posterior inferior line, and anterior line as operator’s discretion. The operator was also able to choose to either perform additional ablations in the superior vena cava or non-PV foci, or conduct the complex fractionated electrogram-guided ablation at his/her discretion. The procedure was considered complete when there was no immediate recurrence of AF after cardioversion with isoproterenol infusion (5 μg/min). If there were AF triggers or atrial premature beats that could be mapped, we carefully mapped and ablated the non-PV foci as much as possible. All RFCA procedures were conducted according to the above-mentioned specific protocol by two operators with over 10 years of experience.

### Follow-up after ablation

Patients visited the outpatient clinic 1, 3, 6, and 12 months after RFCA and every 6 months thereafter. An ECG was obtained at every visit. A 24-hour Holter ECG monitor or an event recorder was worn at 3, 6, 12, 18, and 24 months as a minimum requirement according to the guidelines.[12] Additional ECG, Holter monitor recording, or event monitor recording was obtained when the patient’s symptoms were suggestive of AF recurrence.

### AT mapping in repeat ablation procedure

If the patient maintained sinus rhythm at the beginning of 2nd ablation procedure, we checked block states of previous ablation sites by differential pacing, and then achieved bidirectional blocks by filling the gap. If the initial rhythm was AF, we cardioverted the patient, and repeat above mapping and ablation procedure. In patients with sustaining organized AT, we acquired 3D-activation map first, and defined the conduction gaps of previous ablation sites. We differentiated the mechanism of tachycardia by multi-site entrainment mapping maneuver.[14] If AT morphology changed during RF energy delivery, we mapped multiple ATs one by one consecutively. After AT termination, we evaluated block states of previous ablation sites, and achieved bidirectional blocks. As the final step of repeat ablation, we tested immediate recurrence of AF/AT after cardioversion with isoproterenol infusion as described above, and mapped and ablated the non-PV foci as much as possible.

### Statistical analysis

Continuous variables are presented as means ± standard deviations, and they were compared using Student’s *t*-test. Categorical variables are reported as frequencies (percentages), and they were compared using the Fisher’s exact test. Multivariate logistic regression analysis was performed for the identifiable predictors of recurrence as AT. A p-value <0.05 was considered to be statistically significant. Statistical analyses were performed using SPSS version 23.0.

## Results

### Patient characteristics and recurrence as AT

This study included 521 patients with antiarrhythmic drug-free recurrence after de novo AF ablation. The baseline characteristics of the patients and comparisons according to the type of recurrence are presented in Table 1. The mean age of the patients was 59.3 ± 10.7 years old, 70.8% were male, and 64.1% had paroxysmal AF. Among 521 patients who showed recurrence in the absence of antiarrhythmic drug, 219 patients (42.0%) recurred as AT and remaining 302 patients (58.0%) recurred as AF. Patients with recurrent AT had a higher prevalence of paroxysmal AF (p = 0.007), a lower prevalence of hypertension (p = 0.006), smaller LA volume (p<0.001) and LA volume index (p = 0.002) measured by CT, and higher mean LA bipolar voltage (measured in 355 patients, p<0.001) compared to those with recurrent AF.

**Table 1:** Patient’s characteristics according to type of recurrence (AT vs. AF).

	Overall (n = 521)	Recurrence as AT (n = 219)	Recurrence as AF (n = 302)	p value
Age (years)	59.3 ± 10.7	59.7 ± 10.9	58.9 ± 10.6	0.393
Male	369 (70.8%)	157 (71.7%)	212 (70.2%)	0.770
Paroxysmal AF	334 (64.1%)	155 (70.8%)	179 (59.3%)	**0.007**
BMI (kg/m^2^)	25.1 ± 2.9	24.8 ± 2.8	25.2 ± 3.0	0.119
CHA_2_DS_2_-VASc score	1.80 ± 1.6	1.74 ± 1.59	1.84 ± 1.63	0.493
Heart failure	35 (6.7%)	13 (5.9%)	22 (7.3%)	0.598
Hypertension	244 (46.8%)	87 (39.7%)	157 (52.0%)	**0.006**
Diabetes	85 (16.3%)	37 (16.9%)	48 (15.9%)	0.811
Stroke/TIA	66 (12.7%)	28 (12.8%)	38 (12.6%)	0.999
Vascular disease	83 (15.9%)	32 (14.6%)	51 (16.9%)	0.545
Echocardiographic parameters				
LA size (mm)	41.8 ± 6.2	41.3 ± 6.2	42.1 ± 6.2	0.132
LAVI (ml/m^2^)	37.5 ± 13.2	36.6 ± 13.6	38.2 ± 12.9	0.187
LVEF (%)	63.1 ± 7.8	63.2 ± 6.9	63.0 ± 8.3	0.778
E/Em	10.4 ± 4.0	10.1 ± 3.6	10.6 ± 4.3	0.152
Cardiac CT parameters				
LV volume (mL)	149.9 ± 42.4	141.5 ± 42.6	156.1 ± 41.3	**<0.001**
LA volume index (ml/m^2^)	83.4 ± 24.1	79.2 ± 24.2	86.6 ± 23.7	**0.002**
Venous atrium volume index	27.7 ± 9.3	26.5 ± 9.2	28.5 ± 9.3	**0.023**
LA appendage volume index	6.9 ± 3.0	6.7 ± 2.9	7.1 ± 3.2	0.183
Anterior LA volume index	48.9 ± 15.3	46.0 ± 15.2	51.0 ± 15.1	**<0.001**
Mean LA bipolar voltage (mV) (n = 355)	1.14 ± 0.61	1.27 ± 0.64	1.03 ± 0.56	**<0.001**
Baseline Medications				
ACEi/ARB	177 (34.0%)	65 (29.7%)	112 (37.2%)	0.092
β-blocker	166 (31.9%)	65 (29.7%)	101 (33.6%)	0.392
Statin	154 (29.6%)	61 (27.9%)	93 (30.9%)	0.497

Data are expressed as n (%) or mean ± standard deviation.

ACEi = angiotensin converting enzyme inhibitor; AF = atrial fibrillation; ARB = angiotensin receptor blocker; AT = atrial tachycardia; CT = computed tomography; E/Em = ratio of mitral peak velocity of early filling (E) to early diastolic mitral annular velocity (Em); LA = left atrial; LVEF = left ventricular ejection fraction; TIA = transient ischemic attack.

### Procedural characteristics of de novo ablation according to type of recurrence

**Fig 1:**
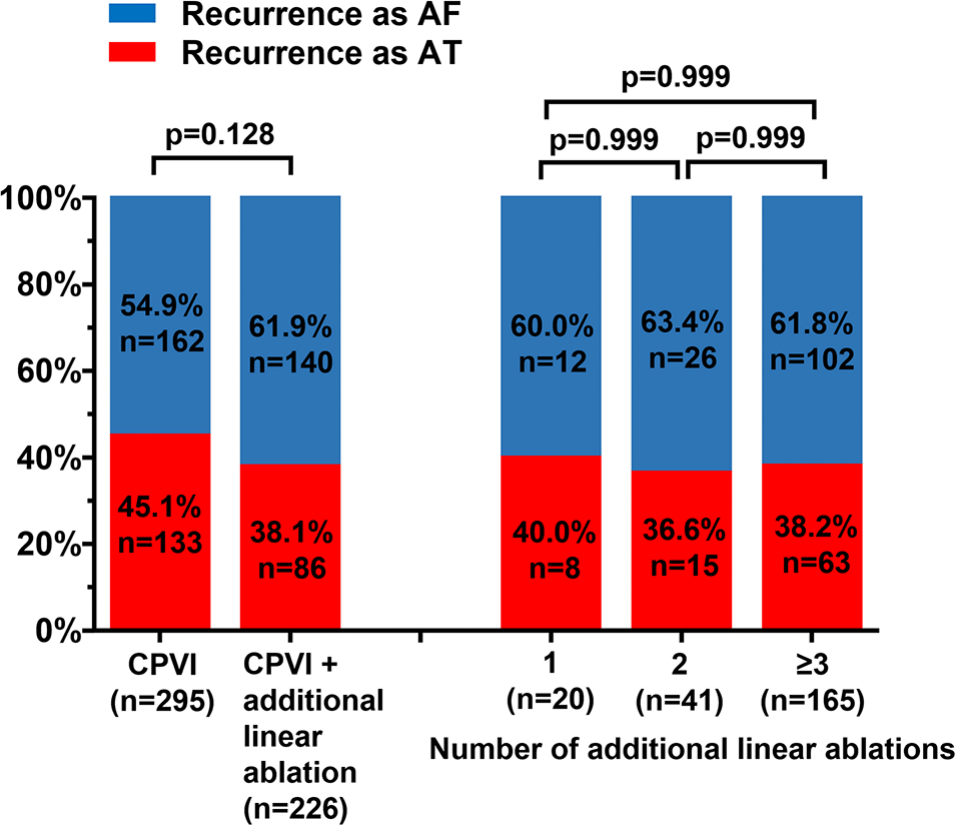
The proportion of recurrence as AT in total recurrence according to lesion set of de novo ablation and number of additional linear ablations. AF = atrial fibrillation; AT = atrial tachycardia; CPVI = circumferential pulmonary vein isolation.

On procedure-related characteristics (Table 2), ablation lesion set, proportion or number of additional linear ablation, and its bidirectional block rates were not significantly different between patients with recurrent AT and those with recurrent AF. However, ablation time (p = 0.046) and procedure time (p = 0.015) was longer in patients with recurrent AT compare to those with recurrent AF. Follow-up duration was 44.7 ± 25.4 months (median: 43 months). Timing of recurrence was 20.8 ± 16.8 months after RFCA (median: 16 months). Early recurrence (recurrence within 3 months) was more frequent in patients with recurrent AT compare to those with recurrent AF (p = 0.012). But late recurrence (recurrence after 3 months) was less observed in patients with recurrent AT (p = 0.002). Fig 1 showed the proportion of recurrence as AT in total recurrence according to lesion set of de novo ablation and number of additional linear ablations. The proportion of recurrence as AT was not different between CPVI only group (n = 295) and CPVI + additional linear ablation group (n = 226). Number of additional linear ablations also did not influence the proportion of recurrence as AT.

**Table 2:** Procedural characteristics of de novo ablation according to type of recurrence (AT vs. AF).

	Overall (n = 521)	Recurrence as AT (n = 219)	Recurrence as AF (n = 302)	p value
Ablation time (sec)	5003.8 ± 1659.5	5174.5 ± 1644.4	4880.1 ± 1662.1	**0.046**
Procedure time (sec)	188.4 ± 53.0	195.0 ± 53.4	183.6 ± 52.3	**0.015**
Additional linear ablation ^a^	226 (43.4%)	86 (39.3%)	140 (46.4%)	0.128
Roof line	222 (42.6%)	86 (39.3%)	136 (45.0%)	0.209
Postero-inferior line	169 (32.4%)	70 (32.0%)	99 (32.8%)	0.850
Anterior line	177 (34.0%)	65 (29.7%)	112 (37.1%)	0.092
Number of additional linear ablation ^a^	1.17 ± 1.42	1.06 ± 1.39	1.25 ± 1.43	0.127
CFAE ablation	30 (5.8%)	13 (5.9%)	17 (5.6%)	>0.999
CTI ablation	480 (92.1%)	207 (94.5%)	273 (90.4%)	0.100
Bidirectional block rates ^b^ of additional linear ablation				
Roof line	158/222 (71.2%)	62/86 (72.1%)	96/136 (70.6%)	0.880
Postero-inferior line	73/169 (43.2%)	27/70 (38.6%)	46/99 (46.5%)	0.346
Anterior line	88/177 (49.7%)	30/65 (46.2%)	58/112 (51.8%)	0.534
Bidirectional block rate of CTI	480/480 (100%)	207/207 (100%)	273/273 (100%)	>0.999
Follow-up duration (months)	44.7 ± 25.4	45.4 ± 25.5	44.3 ± 25.4	0.611
Early recurrence ^c^	339 (65.1%)	156 (71.2%)	183 (60.6%)	**0.012**
Late recurrence ^d^	268 (51.4%)	95 (43.4%)	173 (57.3%)	**0.002**

Data are expressed as n (%) or mean ± standard deviation.

^a^Additional linear ablation includes the following three lesions: roof line ablation, postero-inferior line ablation, and anterior line ablation.

^b^Values are expressed as the number of confirmed bidirectional blocks divided by the number of linear ablations.

^c^Early recurrence: recurrence within 3 months.

^d^Late recurrence: recurrence after 3 months.

AF = atrial fibrillation; AT = atrial tachycardia; CFAE = complex fragmented atrial electrogram; CTI = cavotricuspid isthmus.

### Less remodeled LA rather than linear ablation is associated with AT recurrence

**Fig 2:**
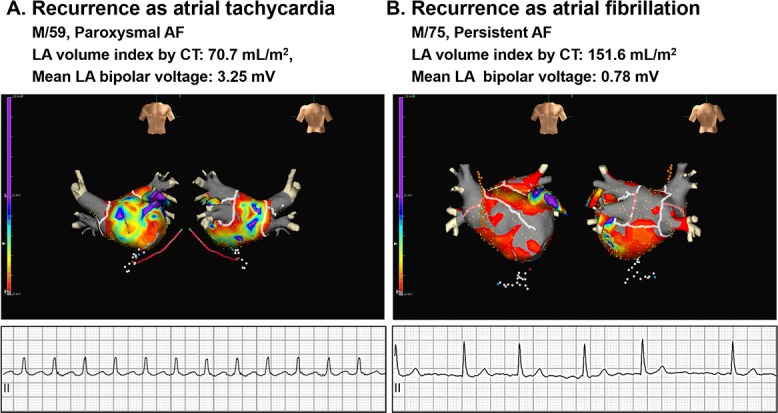
Typical examples of patients with recurrent AT and recurrent AF. **Patients with recurrent AT (A) have a relatively smaller LA volume index and higher mean LA bipolar voltage than patients with recurrent AF (B).** AT = atrial tachycardia; AF = atrial fibrillation; CT = computed tomography; LA = left atrial.

In multivariate logistic regression analysis for recurrence as AT (Table 3), old age (odds ratio [OR] 1.03, 95% confidence interval [CI] 1.01–1.05, p = 0.008), absence of hypertension (OR 0.53, 95% CI 0.35–0.81, p = 0.004), and small LA volume index by CT (OR 0.86 per 10 mL/m^2^, 95% CI 0.78–0.94, p = 0.001) were independently associated with recurrent AT, while additional linear ablation was not (model 1). Among 355 patients with available LA bipolar voltage data (model 2), high LA bipolar voltage (OR 2.03, 95% CI 1.30–3.16, p = 0.002) was also significantly associated with recurrent AT. Fig 2 shows representative examples of LA voltage maps. In consistence, patients with late recurrence after 3 months of RFCA (n = 269), the absence of hypertension (OR 0.47, 95% CI 0.23–0.99, p = 0.049) and high LA bipolar voltage (OR 3.03, 95% CI 1.56–5.88, p = 0.001) were independently associated with recurrent AT (Table 4). However, none of the logistic regression analyses mentioned above showed any relationship between recurrent AT and additional linear ablation.

**Table 3:** Logistic regression analysis for clinical variables predictive of recurrence as AT (analysis including both early ^a^ and late ^b^ recurrence, n = 521).

	Univariate Analysis			Multivariate model 1 ^c^			Multivariate model 2 ^d^		
	OR	95% CI	p value	OR	95% CI	p value	OR	95% CI	p value
Age (per year)	1.01	0.99–1.02	0.393	1.03	1.01–1.05	**0.008**	1.03	1.00–1.05	0.058
Male	0.93	0.63–1.37	0.712						
Paroxysmal AF	1.66	1.15–2.41	**0.007**						
Heart failure	1.25	0.61–2.53	0.544						
Hypertension	0.61	0.43–0.87	**0.006**	0.53	0.35–0.81	**0.004**	0.49	0.29–0.83	**0.007**
Diabetes	0.93	0.58–1.49	0.773						
CHA_2_DS_2_-VASc score	0.96	0.86–1.07	0.492						
LVEF	1.00	0.98–1.03	0.778						
E/Em	0.97	0.93–1.01	0.154						
LA volume index by CT (per 10mL/m^2^)	0.88	0.81–0.95	**0.002**	0.86	0.78–0.94	**0.001**	0.89	0.79–1.00	**0.049**
Mean LA bipolar voltage (per 1mV)	1.97	1.37–2.83	**<0.001**				2.03	1.30–3.16	**0.002**
Ablation time (per 60 sec)	1.01	1.00–1.01	0.063						
Additional linear ablation	0.75	0.53–1.07	0.107						

^a^Early recurrence: recurrence within 3 months.

^b^Late recurrence: recurrence after 3 months.

^c^Model 1: age, sex and clinical variables that had statistical significance for univariate analysis (paroxysmal AF, hypertension, and LA volume index by CT) except mean LA bipolar voltage.

^d^Model 2: variables in the model 1 + LA bipolar voltage (measured in 355 patients).

AF = atrial fibrillation; AT = atrial tachycardia; CI = confidence interval; CT = computed tomography; E/Em = ratio of mitral peak velocity of early filling (E) to early diastolic mitral annular velocity (Em); LA = left atrial; LVEF = left ventricular ejection fraction; OR = odds ratio.

**Table 4:** Logistic regression analysis for clinical variables predictive of recurrence as AT (analysis including only late ^a^ recurrence, n = 269).

	Univariate Analysis			Multivariate model 1 ^b^			Multivariate model 2 ^c^		
	OR	95% CI	p value	OR	95% CI	p value	OR	95% CI	p value
Age (per year)	1.01	0.99–1.04	0.227	1.03	1.00–1.06	0.051	1.03	0.99–1.07	0.077
Male	1.13	0.65–1.97	0.664						
Paroxysmal AF	1.53	0.90–2.59	0.114	1.73	0.95–3.13	0.071			
Heart failure	0.75	0.26–2.20	0.600						
Hypertension	0.73	0.44–1.20	0.211	0.53	0.29–0.98	**0.043**	0.47	0.23–0.99	**0.049**
Diabetes	1.83	0.94–3.56	0.075						
CHA_2_DS_2_-VASc score	1.04	0.88–1.22	0.674						
LVEF	1.02	0.99–1.06	0.255						
E/Em	1.01	0.94–1.08	0.840						
LA volume index by CT (per 10mL/m^2^)	0.97	0.87–1.09	0.619						
Mean LA bipolar voltage (per 1mV)	2.23	1.33–3.75	**0.002**				3.03	1.56–5.88	**0.001**
Ablation time (per 60 sec)	1.01	1.00–1.02	0.055						
Additional linear ablation	1.12	0.68–1.84	0.669						

^a^Late recurrence: recurrence after 3 months.

^b^Model 1: age, sex, paroxysmal AF, hypertension, and LA volume index by CT.

^c^Model 2: variables in the model 1 + LA bipolar voltage (measured in 186 patients).

AF = atrial fibrillation; AT = atrial tachycardia; CI = confidence interval; CT = computed tomography; E/Em = ratio of mitral peak velocity of early filling (E) to early diastolic mitral annular velocity (Em); LA = left atrial; LVEF = left ventricular ejection fraction; OR = odds ratio.

### Repeat ablation findings according to type of recurrence

**Fig 3:**
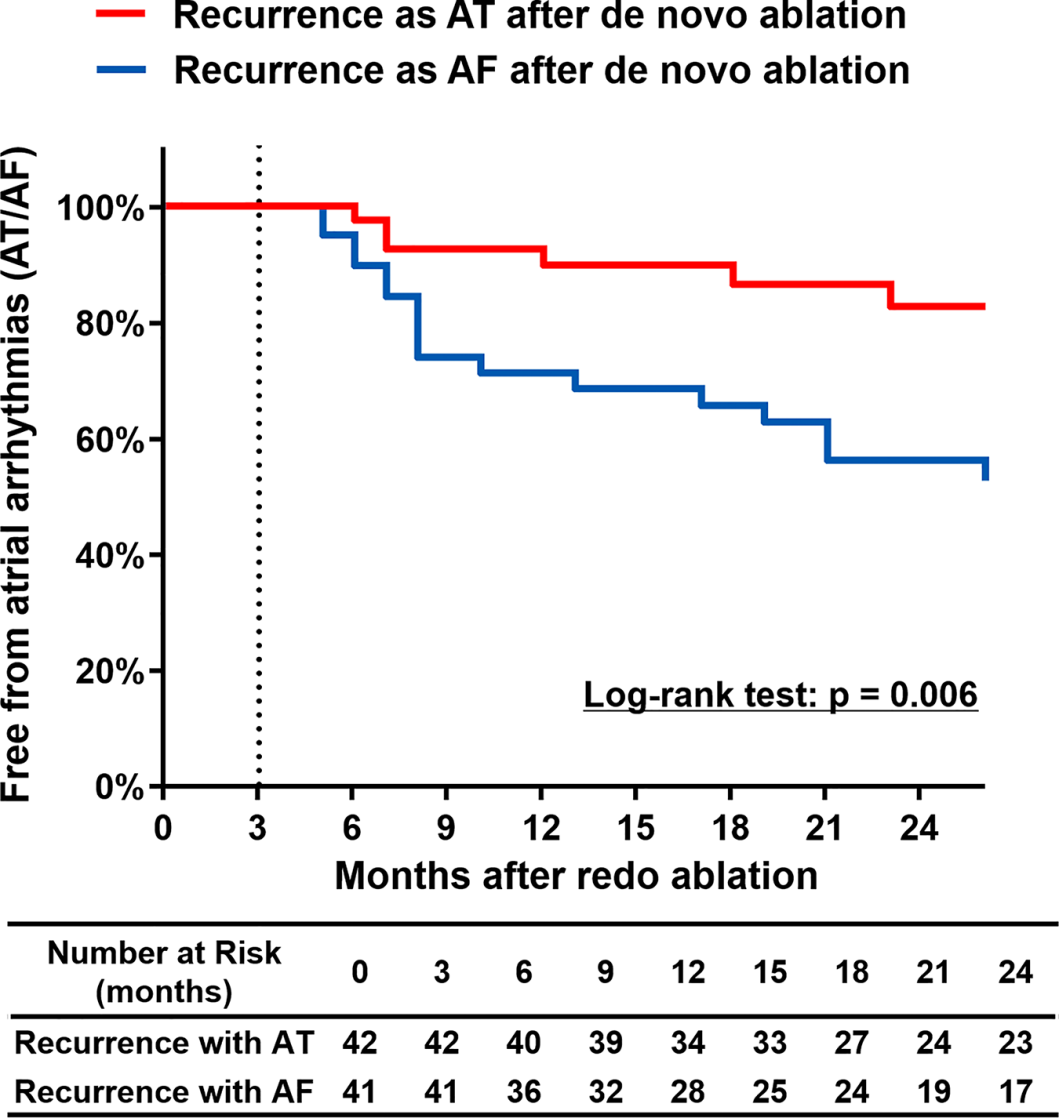
Free from atrial arrhythmias after the redo ablation according to recurrence type of the de novo ablation. AF = atrial fibrillation; AT = atrial tachycardia.

Out of 521 patients, we conducted repeat ablation procedures in 90 patients (17.3%, Table 5). Of these patients with repeat ablation, 50.0% (45) were recurred as AT after de novo procedure. There was no difference in rates of PV reconnection (75.6% vs. 64.4%, p = 0.358), gaps in prior additional linear ablations of LA (90.5% vs. 76.9%, p = 0.269), and gap in prior CTI ablation (31.0% vs. 22.7%, p = 0.468) between recurrent AT group and recurrent AF group. At the beginning of repeat procedure, there were 40 cases in which organized AT was maintained. The mechanisms of ATs were macroreentry in 62.5% (25/40), focal or micro-reentry in 20.0% (8/40), and unmappable due to termination or degeneration to AF in 17.5% (7/40). However, there was no significant difference in frequency of PV reconnection (p = 0.358) and previous linear ablation gap (p = 0.269) between recurrent AT group and recurrent AF group (Table 5). Outcome of repeat ablation was better in patients with recurrent AT compared to those with recurrent AF (p = 0.006; Fig 3).

**Table 5:** Comparison of redo ablation findings according to type of recurrence (AT vs. AF) after de novo AF ablation.

	Overall (n = 90)	Recurrence as AT (n = 45)	Recurrence as AF (n = 45)	p value
Time to the second ablation (months)	25.9 ± 21.1	24.3 ± 22.9	27.5 ± 19.4	0.472
Age	57.0 ± 9.8	56.8 ± 10.2	57.2 ± 9.5	0.823
Male	65 (72.2%)	31 (68.9%)	34 (75.6%)	0.638
Paroxysmal AF	64 (71.1%)	35 (77.8%)	29 (64.4%)	0.245
Number of patients with PV reconnection	63 (70.0%)	34 (75.6%)	29 (64.4%)	0.358
Left superior PV	47 (52.2%)	25 (55.6%)	22 (48.9%)	0.673
Left inferior PV	34 (37.8%)	18 (40.0%)	16 (35.6%)	0.828
Right superior PV	37 (41.1%)	18 (40.0%)	19 (42.2%)	>0.999
Right inferior PV	36 (40.0%)	16 (35.6%)	20 (44.4%)	0.519
Number of reconnected PVs per patient	1.71 ± 1.49	1.71 ± 1.39	1.71 ± 1.59	>0.999
Gap in prior additional linear ablations ^a^	39/47 (83.0%)	19/21 (90.5%)	20/26 (76.9%)	0.269
Roof line	17/47 (36.2%)	7/21 (33.3%)	10/26 (38.5%)	0.768
Posterior-inferior line	25/38 (65.8%)	11/14 (78.6%)	14/24 (58.3%)	0.294
Anterior line	21/35 (60.0%)	7/10 (70.0%)	14/25 (56.0%)	0.704
Gap in prior CTI ablation	23/86 (26.7%)	13/42 (31.0%)	10/44 (22.7%)	0.468
Documented ATs during procedure	40	28	12	
Macro-reentrant	25 (62.5%)	20 (71.4%)	5 (41.7%)	0.091
Gap-related AT ^b^	6 (15.0%)	5 (17.9%)	1 (8.3%)	0.648
CTI-dependent AFL	5 (12.5%)	3 (10.7%)	2 (16.7%)	0.627
Perimitral AFL	8 (20.0%)	6 (21.4%)	2 (16.7%)	>0.999
Roof-dependent reentry	1 (2.5%)	1 (3.6%)	0 (0.0%)	>0.999
Other macro-reentry	5 (12.5%)	5 (17.9%)	0 (0.0%)	0.298
Focal / micro- reentrant	8 (20.0%)	4 (14.3%)	4 (33.3%)	0.211
PV related AT	2 (5.0%)	2 (7.1%)	0 (0.0%)	>0.999
Non-PV trigger AT	6 (15.0%)	2 (7.1%)	4 (33.3%)	0.055
Unmappable due to termination	7 (17.5%)	4 (14.3%)	3 (15.0%)	0.410

Data are expressed as n (%) or mean ± standard deviation.

^a^Values are expressed as the number of non-blocked linear ablations divided by the number of prior linear ablations.

^b^Gap-related AT means that AT re-entry circuit traverses a prior linear ablation line.

AF = atrial fibrillation; AFL = atrial flutter; AT = atrial tachycardia; CTI = cavotricuspid isthmus; PV = pulmonary vein.

## Discussion

### Main findings

In this study, we found that a small LA volume index and high mean LA bipolar voltage, rather than the additional linear ablation in de novo AF ablation, were independently associated with recurrence as AT after catheter ablation of AF. In the sub-analysis of repeat ablation findings, frequency of PV reconnection or previous linear ablation gap was not significantly different between patients with recurrent AT and those with recurrent AF.

### Mechanism of AT after AF ablation

Why do some patients experience recurrence as AT and others as AF? Fibrillation maintenance requires an appropriate critical mass size [8] as well as a short wavelength.[15, 16] The presence of critical mass in atria has been reported in a large animal study,[17] simulation study,[18] and clinical studies.[8] Therefore, less remodeled atrium has low critical mass, appropriate refractoriness, and robust atrial conduction, resulting in lower chance of wavebreak and fibrillatory conduction after CPVI, which favors an AT recurrence pattern. In contrast, patients with significant remodeling and scattered focal atrial scars have a reasonable chance of wavebreak and recurrence as AF, which is triggered from non-PV foci, even after appropriate critical mass reduction.[19] In our study, a large LA volume and low LA voltage, which suggest a more structurally and electrically remodeled LA, were significantly associated with recurrent AF. Although the electrical gap due to incomplete conduction block has been suggested to be one of the important mechanisms of recurrent AT after AF ablation in previous studies,[4, 20] most of those studies were performed after de novo ablation with a non-irrigated tip catheter. However, at present, the use of an irrigated tip catheter has become the standard ablation technique for AF. An irrigated tip catheter can make deeper ablation lesions because it can deliver more energy to the tissue than a non-irrigated tip catheter. Because of this improvement of the catheter, the bidirectional conduction block can be made more effective now and the electrical gap due to incomplete conduction block decreased compared to the past. In the present study, we attempted to generate bidirectional block as much as possible with an irrigated tip catheter in all cases, and the proportion of patients who showed no PV potential at redo ablation was 30.0%. In the redo ablation procedure, only 20.0% of presented ATs were related to prior ablation gaps (15.0% related to prior linear ablation gap, 5.0% related to prior PV ablation gap), but majority (62.5%) of presented ATs were not gap-related AT (macro-reentrant AT unrelated to prior linear ablation lesions: 47.5%, non-PV trigger AT: 15.0%). Remaining 17.5% of ATs were not mappable because they were terminated or changed to AF.

### Disease specific recurrence type after AF ablation

Hypertension is known to increase the risk of AF by about two-fold, and proven to be associated with early and progressive changes in atrial remodeling.[21] The magnitude of structural remodeling of LA is associated with hypertension, and antihypertensive treatments are associated with regression of left ventricular hypertrophy and decreased incidence of new-onset AF episodes.[22, 23] In this study, hypertension was a significant predictor for recurrence as AF rather than recurrence as AT. This might be related to aorto-ventricular and ventriculo-atrial hemodynamic couplings: High central blood pressure results in left ventricular hypertrophy as well as reduced diastolic function,[24] while reduced left ventricular diastolic function increases LA pressure and LA remodeling and reduces LA compliance and voltage.[25] Hypertension-related electroanatomical changes have LA vulnerable to AF rather than AT, when they recur after catheter ablation.

However, the results of current study might be limited to the patients with non-valvular AF with mild to moderate degree of atrial remodeling. In patients with huge atrium with large atrial scar, such as rheumatic AF or valvular AF, most of atrial arrhythmias presented as AT or organized AF. In those patients, slow conduction velocity, short wavelength, and large scar related anatomical obstacles stabilize and organize AT, preventing wavebreak. Therefore, presentation as AT or AF might be determined by atrial pathology and degree of remodeling with bimodal pattern.

### Limitations

The present study has several limitations. First, the study has a retrospective observational design. Second, the recurrence pattern can be affected by the ablation lesion set. Although a consistent ablation lesion set was maintained by two experienced operators, ablation lesion was different between patients with paroxysmal AF and those with persistent AF. Third, since we included patients who experienced recurrence, bidirectional block rates of de novo linear ablations were relatively lower in this study compared to previous reports.[4] In the overall cohort, bidirectional block rates for anterior line and roof line were 64% and 85%, respectively.[26] Forth, a relatively small proportion of patients with recurrence, who were resistant to antiarrhythmic drug therapy, underwent redo ablation. Fifth, in order to include a large number of recurred patients who were not taking antiarrhythmic drugs, we analyzed both early and late recurrence. However, the sub-analysis result of patients with late recurrence (n = 269) was consistent with the result of the overall patients (Table 4). Sixth, LA bipolar voltage maps were analyzable in 68.1% (355 of 521) of included patients.

## Conclusion

Although the electrical gap due to incomplete conduction block was suggested to be an important mechanism of recurrent AT after AF ablation, the degree of LA remodeling is significantly associated with recurrent AT after AF ablation, irrespective of potential ablation gap.
